# Ullmann Coupling Reactions on Ag(111) and Ag(110); Substrate Influence on the Formation of Covalently Coupled Products and Intermediate Metal-Organic Structures

**DOI:** 10.1038/s41598-017-13315-1

**Published:** 2017-11-06

**Authors:** Chris J. Judd, Sarah L. Haddow, Neil R. Champness, Alex Saywell

**Affiliations:** 1 School of Physics and Astronomy, The University of Nottingham, Nottingham, NG7 2RD UK; 2 School of Chemistry, The University of Nottingham, Nottingham, NG7 2RD UK

## Abstract

On-surface reactions based on Ullmann coupling are known to proceed on coinage-metal substrates (e.g. Au, Ag, Cu), with the chemistry of the surface strongly influencing the reaction progression. In addition, the topography of the surface may be expected to affect the local adsorption geometry of the reactants as well as the intermediate and final structures. Here, we investigate the effect of two different surface facets of silver, Ag(111) and Ag(110) on the formation of organometallic and covalent structures for Ullmann-type coupling reactions. Deposition of 4,4”-diiodo-m-terphenyl molecules onto either Ag(111) or Ag(110) surfaces leads to the scission of C-I bonds followed by the formation of organometallic zigzag structures, consisting of molecules connected by coordination bonds to Ag adatoms. The covalently coupled product is formed by annealing each surface, leading to the removal of Ag atoms and the formation of covalently bonded zigzag poly(m-phenylene) structures. Comparisons of the adsorption model of molecules on each surface before and after annealing reveal that on Ag(111), structures rearrange by rotation and elongation of bonds in order to become commensurate with the surface, whereas for the Ag(110) surface, the similarity in adsorption geometry of the intermediate and final states means that no rotation is required.

## Introduction

Over the past decade, there has been great progress in the development of on-surface synthesis of 1D and 2D covalently bonded structures. These have been created through a variety of different strategies^1–7^ facilitating the synthesis of molecular chains,^8–16^ graphene nanoribbons^17,18^, and 2D molecular frameworks^19–24^. Such systems are readily studied by scanning probe microscopies, such as scanning tunnelling microscopy (STM) and atomic force microscopy (AFM), providing real-space imaging with sub-molecular resolution.

**Figure 1:**
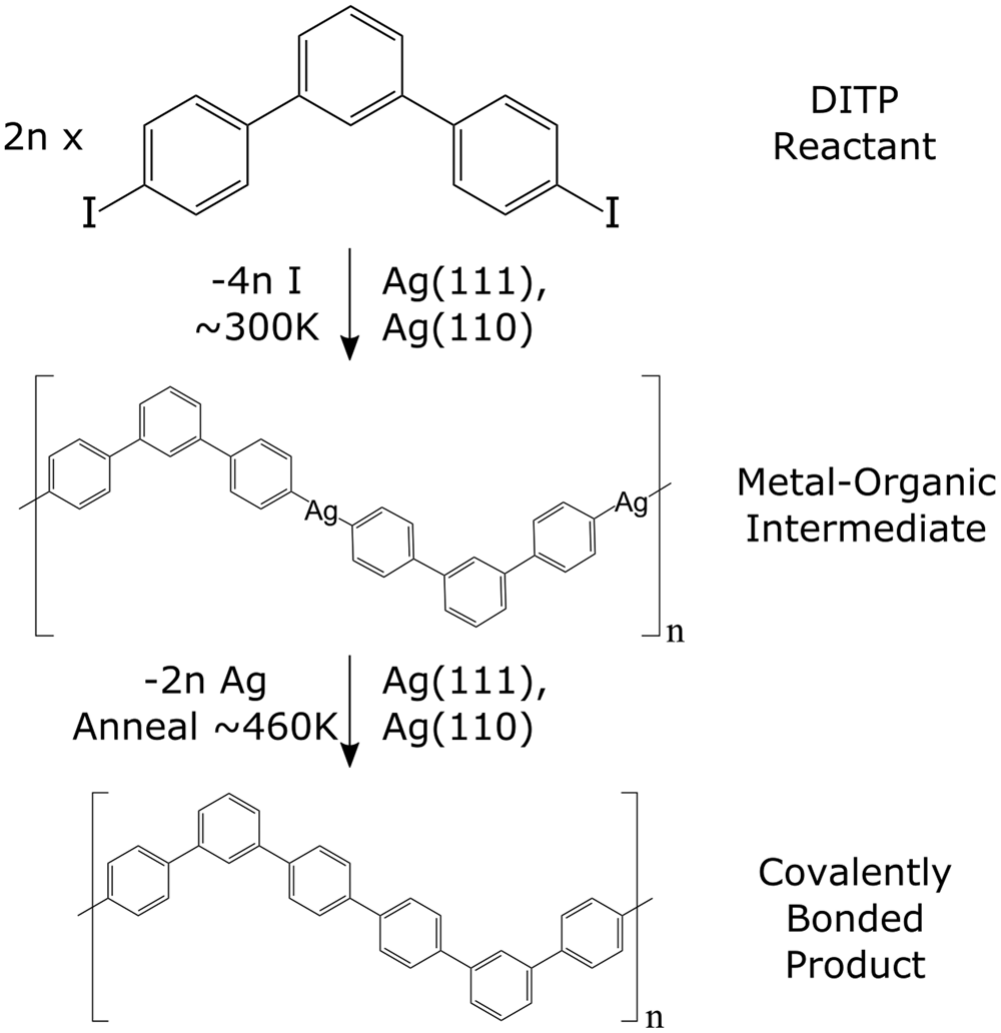
Reaction pathway for 4,4″-diiodo-m-terphenyl (DITP). Deposition onto Ag(111) or Ag(110) surfaces at 300 K, results in iodine dissociation and formation of a metal-organic intermediate structure. After annealing the surfaces at 460 K Ag atoms are eliminated from the intermediate, creating covalent bonds between molecular subunits.

Of particular interest is on-surface synthesis based on the Ullmann coupling reaction^25^. Aryl-halides deposited onto a metal surface undergo dissociation of their halide groups in a reaction catalysed by the metal substrate. The resulting surface-stabilised radicals are then joined via substrate metal adatoms, creating an intermediate metal-organic state. The addition of thermal energy leads to the reductive elimination of the metal atoms, resulting in the formation of covalent bonds between molecular units, shown in Fig. 1 ^3^. This reaction has the advantage of creating covalent bonds between constituent molecules, making structures formed in this way more thermally stable than those formed through hydrogen bonds or van der Waals interactions.

However the mechanistic process by which the intermediate state is formed and converted to the covalent product is still debated. Several studies have attempted to address the problem, investigating the effects of both the bulk chemistry^26^ and topology of the surface^27^, as well as other factors such as the influence of halogens^28,29^ and reaction kinetics^30^, on structure formation. The relationship between the alignment of the intermediate and final structures with the substrate is an additional influential factor to be explored and is investigated here.

Recent studies of 4,4″-dibromo-m-terphenyl on Cu(111)^11,12^ or Cu(110)^13^ have shown the formation of intermediate metal-organic structures at room temperature. After heating the substrates, covalently bonded structures were observed. These structures included zigzag chains and closed hexagonal rings, both before and after annealing. Here, we offer a comparison using a similar, iodine terminated molecule on silver substrates, investigating how the structure of the surface affects the formation of intermediate and final products; with emphasis on the templating effect of the surface.

This investigation used the molecule 4,4″-diiodo-m-terphenyl (DITP), shown in Fig. 1. The molecule consists of three phenylene rings, with the outer aryl groups bonded at the meta positions of the central ring and functionalised with iodine. Deposition of DITP onto silver substrates causes iodine to dissociate from the molecule, followed by the formation of C-Ag-C coordination bonds between molecules. This creates an organometallic intermediate structure. Annealing at 460 K then leads to the removal of linking silver atoms and the formation of a covalently bonded structure. The substrate-molecule system is characterised via STM, and the overlayer structures formed on Ag(111) and Ag(110) are found to be driven by the registry with the surface atoms.

## Results and Discussion

### Metal-Organic Intermediate Structures

Initially, a clean Ag(111) surface was exposed to DITP, resulting in sub-monolayer coverage. Previous studies have shown that similar molecules undergo dissociation of their halide groups upon deposition onto metal surfaces^12^ and it is expected that DITP will behave similarly on silver.

**Figure 2:**
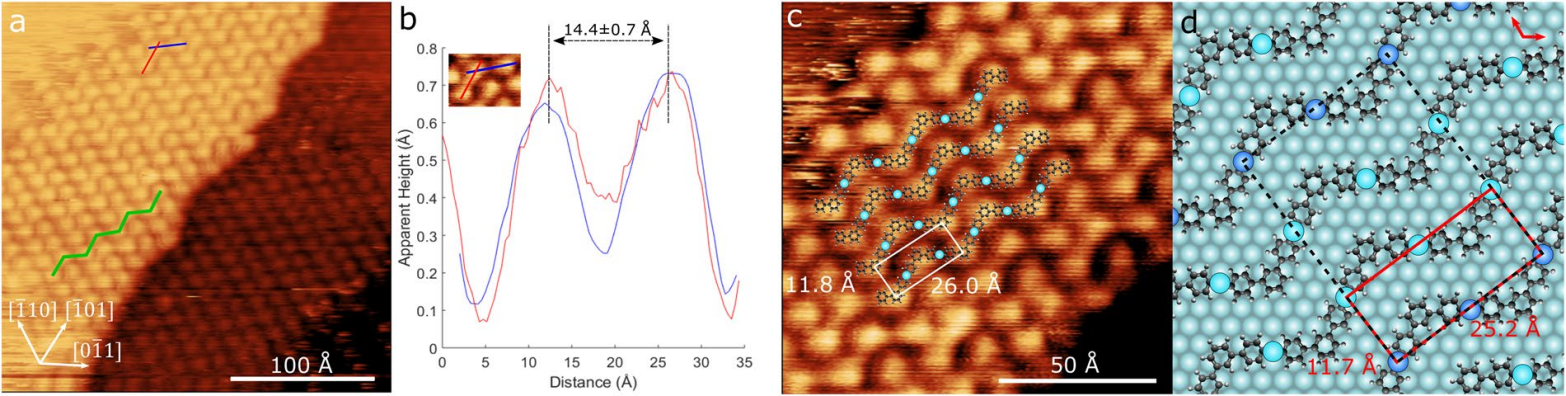
(**a**) STM image of Ag(111) surface after deposition of DITP, including approximate crystallographic directions of the surface lattice. Zigzag structure shown in green (${V}_{sample-bias}$ = −1.5 V, ${I}_{set-point}$ = 5.0 pA). (**b**) Average of 10 line profiles taken along the different directions of the zigzag (red and blue lines shown in (**a**) and inset). Intermolecular separation is consistent with a metal-organic structure. (**c**) Close-up of the DITP island in (**a**) with adsorption model and dimensions overlaid (${V}_{sample-bias}$ = 1.0 V ${I}_{set-point}$ = 5.0 pA). (**d**) Adsorption model for DITP on Ag(111) with overlaid molecular lattice (red), commensurate overlayer structure (black dashed) and surface lattice vectors (top right). Light blue silver adatoms rest in three-fold hollow sites, dark blue in atop sites.

Small islands of molecules were observed to form on the surface, as shown in Fig. 2a. Islands appear as arrays of periodic circular bright features, with each feature attributed to three phenylene rings (DITP after dissociation of iodine), similar to previously observed results^11^. The blurred edges of the island, seen in the top left of Fig. 2a, suggest that molecules are readily diffusing across the surface, with continuous attachment/detachment at the island periphery. A series of images were taken of this area over a 20 minute period and showed the edge of the island growing and shrinking as molecules left then rejoined it. Furthermore, it was observed that STM images acquired at higher current set points ($\mathrm{ > 10}$ pA) could disrupt island structures. This is indicative that the molecules are only weakly bound to the surface and can easily move due to diffusion and tip interactions.

Based on the symmetry of the molecule, the formation of zigzag patterns is expected, highlighted in green in Fig. 2a. To investigate this, several line profiles were aquired over the expected zigzag directions, indicated by red and blue lines in Fig. 2a. The averages of these line profiles are shown in Fig. 2b. Both profiles appear to have the same form as each other, showing two clearly distinguishable peaks, separated by $14.4\pm 0.7$ Å. This is much greater than the expected separation of $12.9$ Å for four covalently linked phenylene rings that are anticipated for the linear component of a covalently coupled structure^24^. This suggests that iodine has dissociated from the DITP molecules and are now linked together with silver to form a metal-organic C-Ag-C bond. The STM data does not exhibit topographic features at the expected location of the Ag atoms. However, this occurence has been observed for similar metal-organic systems^31^, and due to the known convolution of topographic and electronic structure in STM measurements, cannot be used to characterise the height of the Ag atoms relative to the surface.

Islands of molecules were found to consist of a series of parallel zigzag chains, with the long axis length of the chain oriented at approximately ${35}^{\circ }$ to the [$0\overline{1}1$] high symmetry direction of the surface, as shown in Fig. 2a. The zigzag pattern is also measured to have a period of $26.0\pm 0.7$ Å, with the separation of neighbouring chains found to be $11.8\pm 0.7$ Å (Fig. 2c); defining the dimensions of the molecular lattice.

The spatial arrangement of the zigzag pattern and its angle relative to the crystallographic directions of the surface lattice have been used to form an adsorption model for DITP on Ag(111). This is shown in Fig. 2c, overlaid on a close-up of the molecule island seen in Fig. 2a. The proposed molecular arrangement agrees well with the STM images, with circular bright features corresponding to DITP molecules and the regions between them to silver adatoms. Figure 2d shows the adsorption model of DITP on the Ag(111) surface, which is in agreement with estimated lattice directions, with the molecule structure comensurate with the surface. The experimentally measured period and separation of the zigzag pattern ($26.0\pm 0.7$ Å $\times$ $11.8\pm 0.7$ Å) also agrees with the molecular lattice predicted by the adsorption model (based on ideal Ag(111) dimensions and molecular mechanics calculated structure for DITP). However, silver adatoms in different chains are predicted to rest alternately in atop and three-fold hollow sites. Thus, the unit cell for the molecular lattice is different to that of the commensurate overlayer structure. In matrix notation, this unit cell is found to be (4, 6|−2, 11).

In order to investigate the effect of substrate topography on molecular arrangement, the deposition of DITP was repeated onto the Ag(110) surface. This possesses two-fold rotational symmetry, as opposed to the three-fold symmetry of the Ag(111) surface and it is therefore expected that alternative structures will be stabilised.

**Figure 3:**
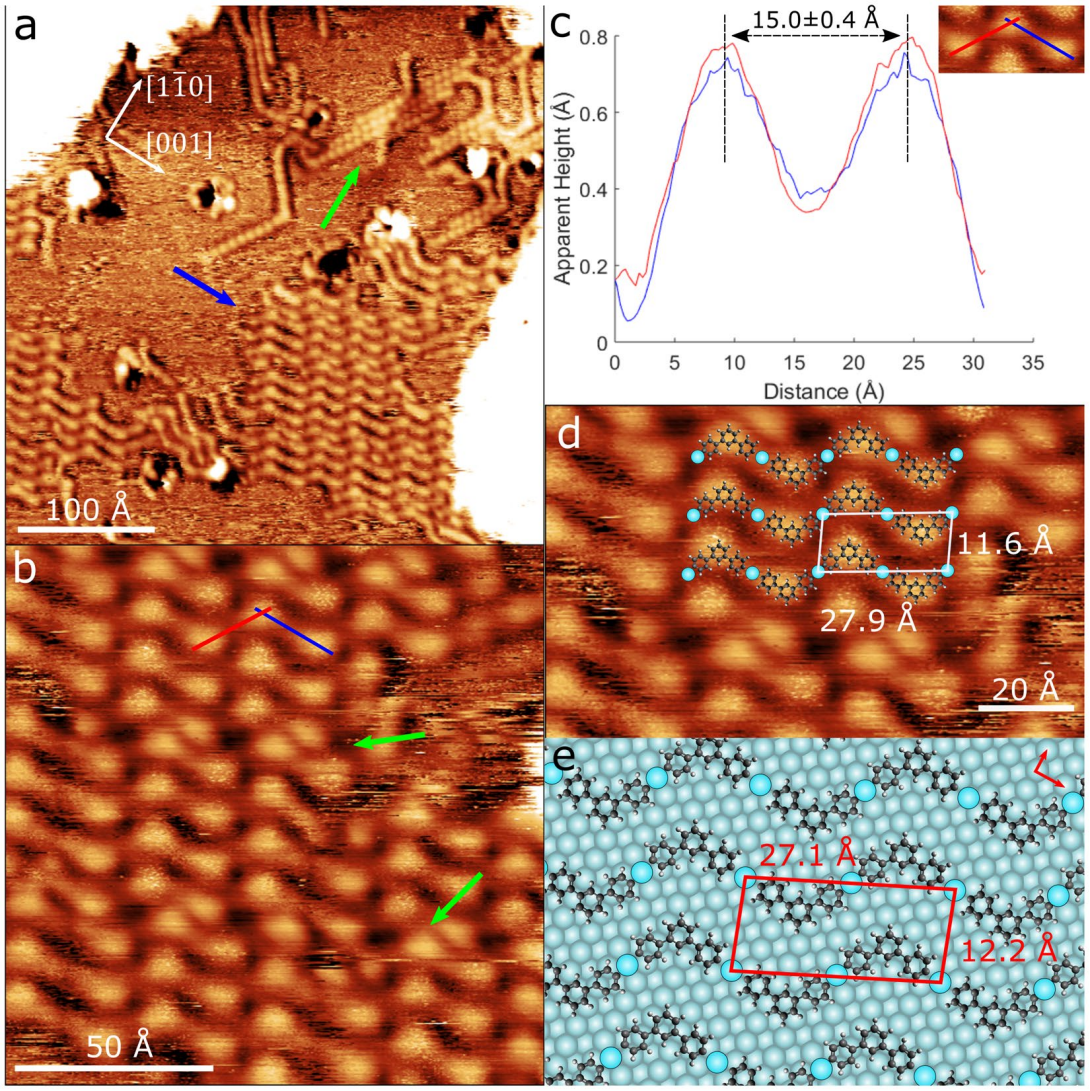
(**a**) STM image of the Ag(110) surface after deposition of DITP. Islands of DITP in a zigzag structure (blue arrow) as well as islands of iodine (green arrow). Lattice directions are overlaid (${V}_{sample-bias}$ = 1.8 V, ${I}_{set-point}$ = 100 pA). (**b**) Close-up of DITP island in (**a**) shown by the blue arrow. Green arrows show areas of disorder in the island (${V}_{sample-bias}$ = 1.0 V ${I}_{set-point}$ = 50 pA). (**c**) Average of 10 line profiles taken along the different zigzag line sections (red and blue lines in (**b**) and inset). Molecule-molecule separation is consistent with proposed metal-organic structure. (**d**) Close-up of metal-organic structure with overlay of proposed model and measured dimensions (${V}_{sample-bias}$ = 1.0 V ${I}_{set-point}$ = 50 pA). (**e**) Adsorption model for DITP on Ag(111) with overlaid molecular lattice (red) and surface lattice vectors (top right).

Figure 3a shows an STM image of the surface after DITP deposition. Bright circular features were observed, connected by dimmer areas to form islands of parallel zigzag chains, similar to those observed on the Ag(111) surface. Two different domains of the zigzag are present, with the direction of the chains approximately $\pm {20}^{\circ }$ to the [$001$] lattice direction (Figure S1). This is expected due to the mirror symmetry about the [$001$] direction and similar results have been observed on Cu(110) substrates^13^.

An additional structure is present on the surface following deposition of DITP (indicated in Fig. 3a with a green arrow). This consists of close-packed rows of circular features separated by $7.2\pm 0.8$ Å. Rows are oriented at ${30}^{\circ }$ to the [$1\overline{1}0$] lattice direction. This arrangement is consistent with features adsorbed at alternate four-fold hollow sites on the Ag(110) lattice (Figure S2). These features were observed to be highly mobile, with the diffusion of mobile species leading to rearrangement of the structure over several STM image cycles. We assign these features to iodine adsorbed to the surface; in agreement with our assertion that iodine has dissociated from the DITP molecules after deposition onto the Ag(110) surface.

Figure 3b shows a close-up of the island in Fig. 3a, indicated by the blue arrow. The island consists of several domains of zigzag chains, slightly displaced relative to each other, leading to the formation of domain boundaries and causing disorder within the island (highlighted in Fig. 3b). Bright circular features at the vertices of the zigzags appear to be DITP after iodine dissociation. These features are separated by $15.0\pm 0.4$ Å. Similar values ($15.7\pm 0.6$ Å^31^) have been observed previously for metal-organic structures on Ag(110), suggesting metal-organic structures containing a single Ag adatom are observed here. Several line profiles were taken of the zigzag pattern and their averages are shown in Fig. 3c. The overall shape of the line profiles and the peak separation are very similar to that seen on Ag(111), suggesting that deiodinated DITP molecules are forming metal-organic bonds with silver adatoms.

An adsorption model for this arrangement of molecules is proposed based on measurements of feature dimensions and angles relative to the silver surface (Fig. 3d). The model agrees well with the STM data, with DITP and silver adatoms corresponding to circular bright features and the areas linking them respectively. The period of the zigzag chain was measured to be $27.9\pm 0.4$ Å and the separation of chains $11.6\pm 0.4$ Å (Fig. 3d). The separation of zigzag chains is consistent with observations on the Ag(111) surface ($11.8\pm 0.7$ Å), whereas the zigzag period is noticeably larger, by approximately $2$ Å. The differences in size of the molecular lattices on each surface can most likely be attributed to elongation of the C-Ag bonds between molecules, facilitating a better registry with the different surface lattices. This difference in the molecular lattice is also greater than that observed for zigzag structures formed from similar molecules on Cu(111)^11^ and Cu(110)^13^, where the difference was found to be ∼1.5 Å. This highlights the effects of different surfaces on structure formation.

Figure 3e shows the adsorption model for DITP on the Ag(110) surface. It can be seen that the model is commensurate with the surface, with the silver adatoms resting in four-fold hollow sites and DITP spaced evenly between them. The measured separation and period for the zigzag arrangement also agrees well with the predicted dimensions of the molecular lattice. With respect to the Ag(110) lattice, in matrix notation, the unit cell of the commensurate overlayer structure is found to be (6, 4|−1, 4).

### Covalently Coupled Products

**Figure 4:**
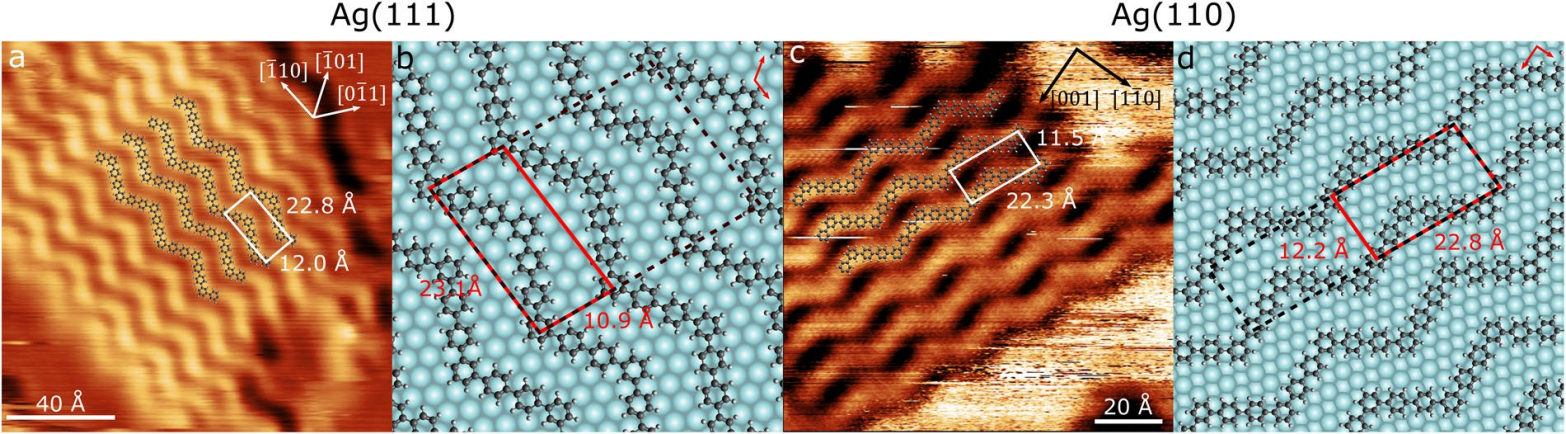
(**a**) STM image of the annealed Ag(111) surface seen in Fig. 2. Overlaid are the DITP adsorption model, structure dimensions and lattice vectors of the surface. (${V}_{sample-bias}$ = −1.0 V ${I}_{set-point}$ = 5.0 pA). (**b**) Adsorption model for DITP on Ag(111) after annealing at 460 K with overlaid molecular lattice (red), commensurate overlayer structure (black dashed) and surface lattice vectors (top right). (**c**) STM image of annealed Ag(110) surface seen in Fig. 3 with overlaid adsorption model, structure dimensions and surface lattice vectors (${V}_{sample-bias}$ = 1.5 V ${I}_{set-point}$ = 5.0 pA). (**d**) Adsorption model for DITP on Ag(110) after annealing at 460 K with overlaid molecular lattice (red), commensurate overlayer structure (black dashed) and surface lattice vectors (top right).

Following previous methodologies^13,14^, the covalently coupled products were obtained by an additional thermal annealing step, heating samples to 460 K. Examples of the resulting structures for the Ag(111) and Ag(110) surfaces are shown in Fig. 4a and c, respectively. In both cases, islands of molecules consisting of parallel chains in a clear zigzag pattern were observed. The zigzag structures exhibit a constant apparent height along their length, with no individually resolved features (as opposed to the clearly distinguishable circular features of the metal-organic structure observed prior to heating). Similar to the structures observed prior to annealing, two different zigzag domains were present on the Ag(110) surface, oriented at $\pm {20}^{\circ }$ to the [$001$] surface lattice direction. The period of the zigzag was found to be $22.3\pm 0.5$ Å for the Ag(111) surface and $22.8\pm 0.5$ Å for Ag(110), smaller than that observed for the metal-organic structures ($26.0\pm 0.7$ Å on Ag(111) and $27.9\pm 0.4$ Å on Ag(110)). The separation between the chains were found to be $12.0\pm 0.5$ Å and $11.5\pm 0.5$ Å for Ag(111) and Ag(110) respectively, which are consistent with chain separation measurements before annealing.

The very similar values for the repeat unit along the zigzag domain on both annealed substrates suggests the same structure has formed on both surfaces. Additionally, both repeat units are significantly smaller than those observed for each structure prior to annealing. Due to the lack of resolvable features, and the apparent shortening of chains, it is suggested that silver atoms have been eliminated from the DITP monomer units, leading to the formation of covalent bonds. Adsorption models for the annealed samples are proposed and are shown overlaid on examples of the zigzag islands in Fig. 4a and c. In both cases, the modelled molecular chains agree well with STM images of the structures.

Figure 4b shows the proposed adsorption model on the Ag(111) lattice. As for the sample prior to annealing, the vertices of the zigzag on different chains alternate between atop and three-fold hollow sites. The model agrees well with the measured period and separation of the zigzag. In matrix notation, the unit cell for the commensurate overlayer structure is found to be (8, 0|8, 5).

It is interesting to note that for the commensurate overlayer structure, zigzag chains are oriented differently before and after annealing the surface. Prior to annealing, the chain is ∼30° to the [$0\overline{1}1$] direction, whereas after annealing, it runs parallel to this crystallographic axis. This shows that the zigzag structures have undergone a significant rearrangement, indicating that the mechanism for covalent coupling is not simply removal of Ag atoms but significant molecular displacement is also involved. This structural alteration is likely to be driven by the reduction in surface free energy associated with maintaining a commensurate structure in both the metal organic intermediate and covalently bonded states. However, this is in contrast to previous measurements of zigzag structures on Cu(111)^11^, where no significant reorientation is observed, showing the influence of surface chemistry on structure formation.

The adsoprtion model for DITP chains on the Ag(110) surface is shown in Fig. 4d. Molecules are aligned to the lattice such that one arm of the zigzag is almost parallel (<5°) to the [$001$] direction. This is a similar arrangement to the model before annealing, and shows molecules have not needed to reorientate significantly after heating to be commensurate with the surface, in contrast to the large change in orientation observed for the Ag(111) surface. Similar results have been observed previously for Cu(110) annealed zigzag structures^13^. The two centre rings on the parallel arms of the zigzag rest alternately in four-fold hollow and long bridge sites on the lattice. In matrix notation, the unit cell of the commensurate overlayer structure was found to be (10, −7|1, 4).

It should be noted that a small number of hexagonal structures were also seen on the Ag(111) surface (Figure S3). However these highly mobile features are difficult to image. Similar structures have been observed previously on ($111$) metal surfaces^11,32^. The study on a silver surface however was performed at 78 K or lower, suggesting that there may be more hexagonal structures on the surface, but they are diffusing too rapidly to be observed at room temperature.

Table 1 shows a comparison of the measured directions for the observed structures. For both substrates, before and after annealing, the separations between zigzag chains (inter-row separations) are very consistent. This is possibly due to iodine atoms resting between the chains, causing them to maintain a constant separation. Similar observations were made on both Cu(111)^12^ and Cu(110)^13^ where it was proposed that assembly of islands from the zigzag chains was assisted by bromine adatoms forming hydrogen bonds with the chains, helping to stabilise the structure. Additionally, both the Ag(111) and Ag(110) annealed samples have very similar periods, whereas prior to annealing significant variation between zigzag period for Ag(111) and Ag(110) was observed. This is likely due to the limited variation in lengths achievable by the C-C bonds in the covalently bonded structures formed after annealing, in contrast to the greater degree of flexibility between DITP molecules, facilitated by metal-organic bonds. This variation in bond length allows molecules to adopt energetically favourable positions, potentially allowing a greater diversity of structures, dependent on the surface chosen.

**Table 1:** Comparison of STM Image Measurements and Adsorption Model Unit Cells.

	experimental data^*a*^		adsorption model^*b*^	
	zigzag period (Å)	inter-row separation (Å)	zigzag period (Å)	inter-row separation (Å)
Ag(111)	$26.0\pm 0.7$	$11.8\pm 0.7$	$25.2$	$11.7$
Ag(110)	$27.9\pm 0.4$	$11.6\pm 0.4$	$27.1$	$12.2$
annealed Ag(111)	$22.8\pm 0.5$	$12.0\pm 0.5$	$23.1$	$10.9$
annealed Ag(110)	$22.3\pm 0.5$	$11.5\pm 0.5$	$22.8$	$12.2$

^*a*^Values for zigzag dimensions are based on repetitions seen in structures in the STM images.

^*b*^Adsorption model distances are calculated based on the unit cells of the silver lattice and molecular overlayer.

## Conclusions

The on-surface Ullmann-type coupling reaction has been demonstrated for 4,4″-diiodo-m-terphenyl (DITP) on Ag(111) and Ag(110) surfaces. Deposition onto these surfaces at room temperature causes dissociation of iodine, followed by the formation of metal-organic structures, via bonding with Ag adatoms on the surface. In both cases, islands of parallel zigzag chains are formed. Adsorption models for molecules on each substrate were proposed and showed C-Ag-C bonds stretch to allow the formation of commensurate structures on Ag(111) and Ag(110), producing different sized structures on each surface.

Annealing of the surfaces at 460 K results in removal of Ag adatoms from the structures and the formation of covalently-bonded continuous zigzag chains. It was found that on Ag(111) the resulting poly(m-phenylene) chains undergo a rotation of approximately 30° relative to the surface lattice, in order to achieve commensurability with the surface. This is in contrast to the Ag(110) surface where no rotation was observed, or required, to achieve commensurability. Our results clearly demonstrate the influence of substrates on the formation of covalently-coupled structures on surfaces, and that the relationship between orientation of the intermediate and final state may assist transit along the reaction pathway.

## Experimental Methods

Experiments were performed with an Omicron variable temperature STM system at base pressures below ${10}^{-9}$ mbar. Images were acquired in constant current mode on samples held at 300 K using electrochemically etched Tungsten tips (coated in silver during tip optimisation by controlled indentation into the surface). The two substrates employed in these experiments were a Ag(111) on mica sample (Georg Albert PVD GmbH) and a Ag(110) single crystal (MaTecK GmbH). Both samples were prepared by Argon ion sputtering ($0.8\,keV$, ${I}_{sample}=5.0\,\mu A$) for 10 minutes followed by annealing at 400 °C for 30 minutes. DITB was prepared using previously published procedures^33^ and deposited onto surfaces by exposing them for 5 minutes to the flux from a knudsen cell, heated to 130 °C. Molecular mechanics simulations were performed using Avagadro^34^ with the Universal Force Field (UFF)^35^. These simulations, along with crystal dimensions and experimental measurements of the molecular overlayer, were used to produce adsorption models for 4,4″-diiodo-mterphenyl on Ag(111) and Ag(110).

### Data availability

Supplementary information available on: Details of molecular overlayer alignment to the substrate; Analysis of iodine based structures; Discussion of additional reaction path; This material is available free of charge via the internet at: https://www.nature.com/srep/.

The experimental data on which this work is based, including STM image files, may be found at:

http://dx.doi.org/10.17639/nott.325.

## Electronic supplementary material

Supplementary Information
