# Supplementary material for: Ullmann Coupling Reactions on Ag(111) and Ag(110); Substrate Influence on the Formation of Covalently Coupled Products and Intermediate Metal-Organic Structures
Source: Sci Rep. 2017 Nov 6;7:14541. doi: 10.1038/s41598-017-13315-1 (PMC5674052; doi:10.1038/s41598-017-13315-1)
Supplement: Supplementary file 1 — Supplementary Information [file 41598_2017_13315_MOESM1_ESM.pdf]

Ullmann Coupling Reactions on Ag(111) and Ag(110); Influence of Substrate on the Formation of Covalently Coupled Products and Intermediate Metal-Organic Structures

Chris J Judd,[†] Sarah L Haddow,[‡] Neil R Champness,[‡] and Alex Saywell*[†]

† School of Physics and Astronomy, The University of Nottingham, Nottingham, NG7 2RD, UK

‡School of Chemistry, The University of Nottingham, Nottingham, NG7 2RD, UK

*E-mail: Alex.Saywell@nottingham.ac.uk

Contents

1. Details of Molecular Overlayer Alignment to Substrate 2
2. Analysis of Iodine Based Structures 4
3. Discussion of Additional Reactant Products 4
Bibliography 5

1. Details of Molecular Overlayer Alignment to Substrate

Figure S1 shows overview images for the Ag(111) and Ag(110) surfaces with deposited 4,4''-diiodo-m-terphenyl, before and after annealing. These images were used to determine the directions of the underlying surface lattice. This was achieved by identifying the direction of step edges in each image. For Ag(111), there are typically three step edge directions, each indicating approximately the directions of the three high symmetry directions of the surface. For the Ag(110) surface, the majority of straight step edges follow the [110] direction,[S1] and this was used to determine the lattice directions for the acquired scanning tunnelling microscopy (STM) images. Additionally, figure S1 shows the directions of zigzag chains in different islands (blue arrows) for each substrate. These directions were used to calculate the angles of each zigzag chain, relative to the surface, as discussed in the main manuscript.

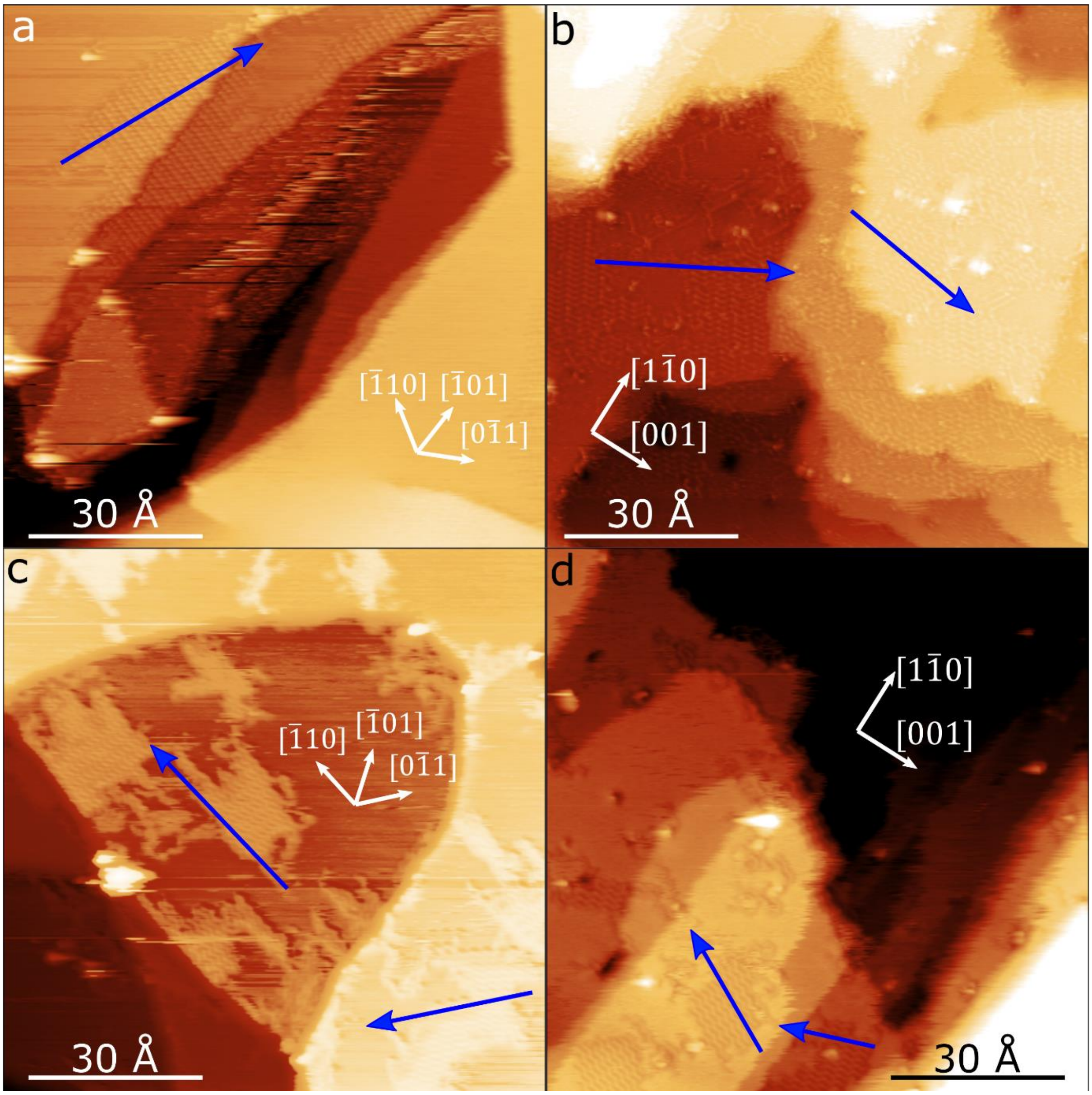

Figure S1: STM images showing directions of zigzag chains (blue arrows) relative to surface lattice vectors. In each case, surface lattice directions were obtained from the directions of flat step edges and are shown in white. (a) Ag(111) surface before annealing ($V_{sample-bias}$ = -1.5 V $I_{set-point}$ = 5.0pA). (b) Ag(110) surface before annealing. Two differently oriented domains are observed at ±20° to the [001] lattice direction ($V_{sample-bias}$ = 1.8 V $I_{set-point}$ = 100pA). (c) Ag(111) surface after annealing. Two different island domains are observed with chains parallel to one surface lattice direction ($V_{sample-bias}$ = -1.0 V $I_{set-point}$ = 5.0pA). (d) Ag(110) surface after annealing. Two differently oriented domains are observe at ±20° to the [001 lattice direction ($V_{sample-bias}$ = 1.5 V $I_{set-point}$ = 5.0pA).

2. Analysis of Iodine Based Structures

Figure S2a shows a close up of the iodine islands observed on the Ag(110) surface. The rows of these islands (indicated by the blue arrow) are oriented at 30 to the [110] lattice direction and each feature is separated by 7.2 ± 0.8 Å. From this information the adsorption model shown in figure S2b is proposed with iodine atoms resting in alternate four-fold hollow sites. The overlayer unit cell in matrix notation is given by (2,0|1,2).

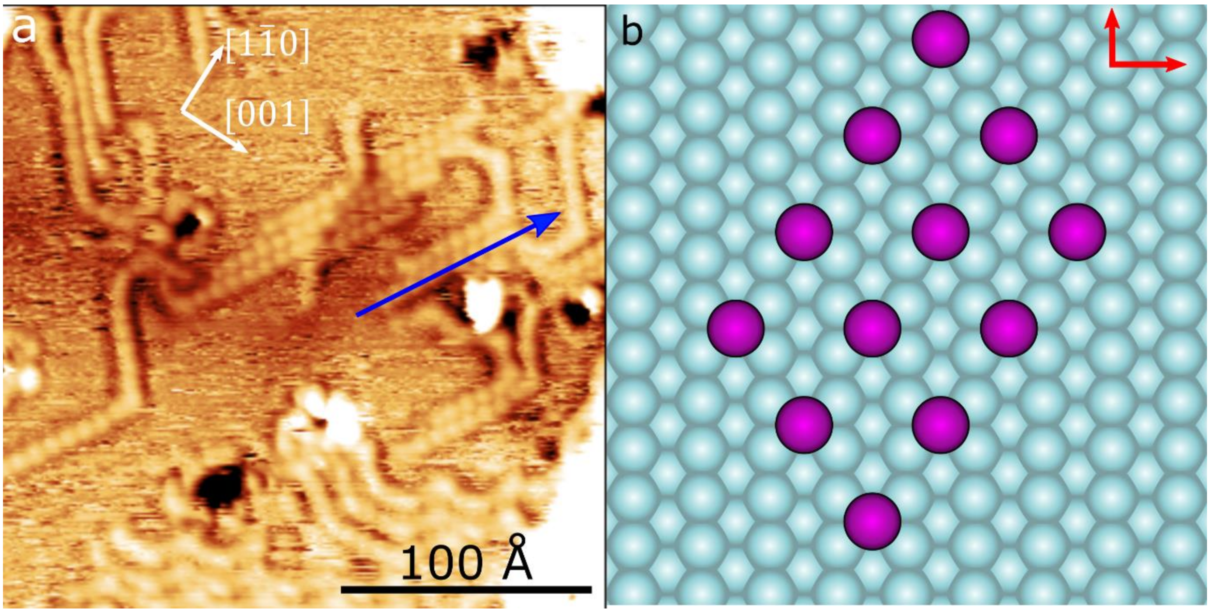

Figure S2: (a) STM image of an island of iodine on the Ag(110) surface before annealing with lattice directions overlaid ($V_{sample\text{-}bias}$ = 1.8 V $I_{set\text{-}point}$ = 100pA). (b) Proposed adsorption model for iodine on Ag(110) surface lattice, based on observed separation of features and their orientation relative to surface lattice directions.

3. Discussion of Additional Reactant Products

Figure S3a shows an island structure on the annealed Ag(111) surface consisting of an array of bright circular features, separated by 32 ± 2 Å with rows at an angle of ~60° relative to each other. These features are attributed to an island of close packed hexagonal structures imaged in an inverted contrast mode, with circular bright features corresponding to the centres of each hexagon. Figure S3b shows a close-up of the same area, with the image colouring inverted. Hexagonal structures can clearly be seen at the edges of the island. Additional structures such as this may be present elsewhere, however, hexagonal structures were only observed once on the Ag(111) surface after annealing and not at all on Ag(110).

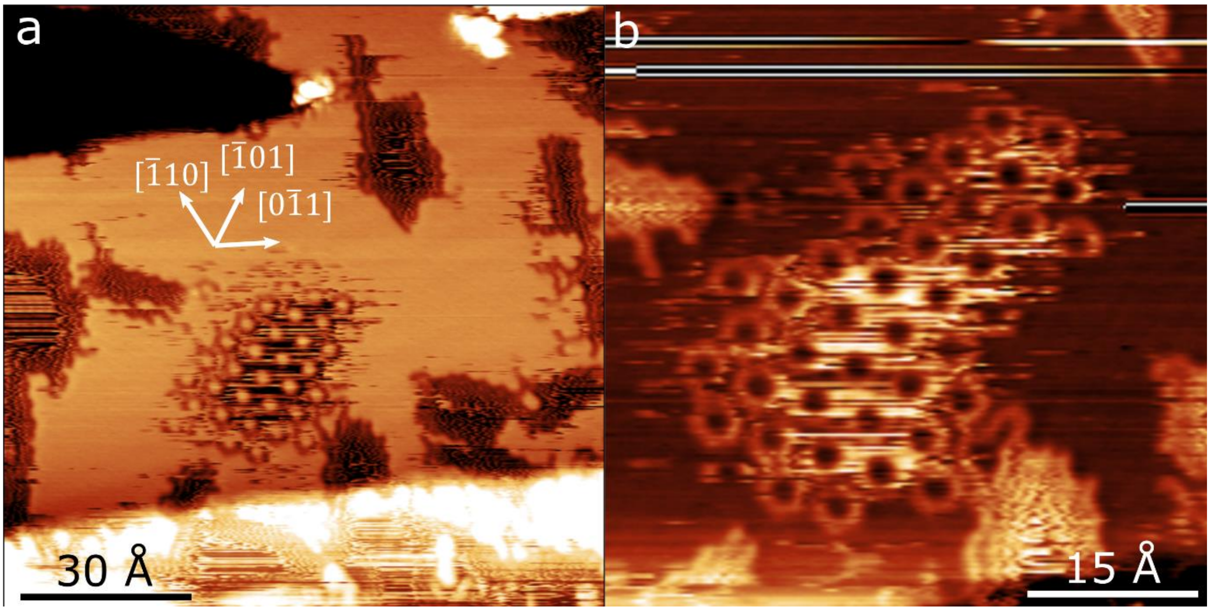

Figure S3: (a) STM image showing the hexagonal structures observed on the Ag(111) surface after annealing at 460K. Surface lattice directions are overlaid ($V_{sample\text{-}bias}$ = -1.0 V $I_{set\text{-}point}$ = 5.0pA). (b) Close-up of island of hexagonal structures. Image colouring is inverted for clarity ($V_{sample\text{-}bias}$ = -1.0 V $I_{set\text{-}point}$ = 5.0pA).

Bibliography

S1. Koch, R., Schulz, J. J. & Rieder, K. H. Scanning tunneling microscopy artifact and real structure: Steps of Ag(110). *EPL,* **48,** 554 (1999).
